# Scaling up the fabrication of wafer-scale Ni-MoS_2_/WS_2_ nanocomposite moulds using novel intermittent ultrasonic-assisted dual-bath micro-electroforming

**DOI:** 10.1016/j.ultsonch.2023.106359

**Published:** 2023-03-07

**Authors:** Tianyu Guan, Yuanzhi Lu, Xinhui Wang, Michael D. Gilchrist, Fengzhou Fang, Nan Zhang

**Affiliations:** a Centre of Micro/Nano Manufacturing Technology (MNMT-Dublin), School of Mechanical & Materials Engineering, University College Dublin, Dublin 4, Ireland; b State Key Laboratory of Precision Measuring Technology and Instruments, Laboratory of Micro/Nano Manufacturing Technology (MNMT), Tianjin University, Tianjin 300072, China

**Keywords:** Ultrasonication, Electroforming, Friction, Mould, Nanosheets

## Abstract

In the scale-up fabrication process for electroformed Ni-MoS_2_/WS_2_ composite moulds, the formulation of nanosheets is critical, since the size, charge, and their distribution can largely affect the hardness, surface morphology and tribological properties of the moulds. Additionally, the long-term dispersion of hydrophobic MoS_2_/WS_2_ nanosheets in a nickel sulphamate solution is problematic. In this work, we studied the effect of ultrasonic power, processing time, surfactant types and concentrations on the properties of nanosheets to elaborate their dispersion mechanism and control their size and surface charge in divalent nickel electrolyte. The formulation of MoS_2_/WS_2_ nanosheets was optimized for effective electrodeposition along with nickel ions. A novel strategy of intermittent ultrasonication in the dual bath was proposed to resolve the problem of long-term dispersion, overheating, and deterioration of 2D material deposition under direct ultrasonication. Such strategy was then validated by electroforming 4-inch wafer-scale Ni-MoS_2_/WS_2_ nanocomposite moulds. The results indicated that the 2D materials were successfully co-deposited into composite moulds without any defects, along with the mould microhardness increasing by ∼2.8 times, the coefficient of friction reducing by two times against polymer materials, and the tool life increasing up to 8 times. This novel strategy will contribute to the industrial manufacturing of 2D material nanocomposites under ultrasonication process.

## 1 Introduction

Precision replication of micro/nano scale features on a polymeric substrate using mass production technologies, e.g., micro injection moulding, roll-to-roll micro hot embossing/nano imprinting, is critical for a variety of applications including microfluidic devices, micro-optics, photonics, and anti-stick surfaces [1], [2], [3], [4], [5]. However, when separating solidified polymers from micro-scale moulds, due to the large surface-to-volume ratio, it is the surface and interface between the mould and polymer that largely governs the integrity of the moulded micro structures [6]. For example, adhesion between the polymer and mould due to interfacial chemical reactions and molecular attraction can easily cause surface damage or distortion of a structure. Additionally, mechanical interlocking from the mould surface topography can cause friction between the polymer and mould, leading to similar damage and plastic deformation of micro structures on polymer workpieces [6], [7], [8]. The deposition of nanomaterials such as MoS_2_/WS_2_ nanosheets into a nickel mould by means of micro-scale electroforming has resulted in improved microhardness and lubricating properties, which have enhanced mould wear resistance and facilitated the ease of demoulding of micro-scale features [9], [10]. Incorporating micro/nanomaterials in a nickel composite mould was shown to reduce the friction-induced surface damage and adhesion-induced structure distortion [10], [11], which are the main causes of demoulding defects in micro injection moulding and micro hot embossing [6], [12]. MoS_2_/WS_2_ nanosheets have layered molecular structures and weak Van der Waals bonds between the adjacent sulphur atoms, which lead to interlamellar mechanical weakness [10], [13], [14]. It is this structural property that provides MoS_2_/WS_2_ nanosheets with excellent self-lubricating properties, making them suitable for use as additives that can be dispersed and added to composite materials for improving hardness and lubrication [15], [16], [17], [18]. However, the strong Van der Waals forces among these nanosheets and their hydrophobic surface properties mean they have poor solubility and dispersibility in an electrolyte and tend to form large aggregates in a plating bath [13], [14], [19]. This constitutes a significant challenge to uniformly dispersing nanosheets in the electrolyte bath and to incorporating the content of the nanosheets in the metal deposits, especially during the scale-up fabrication of composite moulds. Insufficient agitation or unstable dispersion may result in roughened, fragile or porous deposits [19], [20], which would further affect the quality of the composite mould, since low surface roughness (typically lower than 100 nm in Ra) is required for precision replication of polymer workpieces [10].

Studies regarding the electrodeposition of metal nanocomposites based on nickel-MoS_2_/WS_2_ often involve agitation in a magnetically-stirred beaker with the use of surfactants, which is easily achieved in a laboratory [19], [20]. In our previous study, a Ni/WS_2_ composite mould was electroformed in a magnetically-stirred beaker with a total surface area of ∼4 cm^2^, which is too small for most microfuidic applications [14]. Additionally, that particular setup was unsuitable for scaling up the fabrication of larger nanocomposite moulds, where a four-inch to six-inch wafer is commonly used as a cathode in typical micro electroforming [20]. In the scale-up of micro-electroforming a four-inch wafer-scale nickel composite mould, the aggregation of MoS_2_/WS_2_ nanosheets in a large amount of nickel sulphamate solution (∼1 L) is problematic. Different from composite coatings, the composite mould has a thickness of ∼500 µm, which requires long-term stability of nanosheet dispersion in the electrolyte solution. During the experiments, MoS_2_/WS_2_ aggregates were found in the electrolyte solution during the co-deposition process, even with the aid of surfactants and pre-ultrasonic treatment, which was also highlighted as a challenging problem to be solved in other studies [13], [16], [21]. Ultrasonic waves have been used as an effective tool to exfoliate bulk layers of nanomaterials such as in the synthesis of nanomaterials [22], catalysis [23], and electrodeposition [24]. The assistance of ultrasound has proven to be effective in the co-deposition of micro/nanoparticles and nickel in terms of improving the dispersion of particles in the electrolyte solution and enhancing the content of uniformly-dispersed particles that are incorporated in the metal composites [25]. The use of an ultrasonic bath has been found to be effective in dispersing particles during electrodeposition to fabricate Ni-diamond [26] and Ni-MoS_2_ composite coatings [27]; however, these coatings had a small surface area (∼1 cm^2^) and thickness, requiring short deposition times (less than one hour), which is not sufficient for fabricating large-scale composite moulds. Ultrasonic probes have also been used during electroforming to disperse Al_2_O_3_ nanoparticles in nickel Watts bath to produce Ni-Al_2_O_3_ composite coatings with a surface area of 3 cm^2^ and a deposition time of 30 min [28]; however, the application of ultrasonication in the electroplating bath may interfere with the electrochemical reactions in the working electrode. Most importantly, the composite coatings fabricated using these ultrasonic-assisted methods had high surface roughness, which reduced the deposit quality and increased the friction due to high mechanical interlocking. In the fabrication of composite moulds, a mould with high surface roughness would suffer significant friction-induced wear during demoulding, and the surface quality of the replicated polymer workpieces would be poor due to high adhesion and friction forces at the mould-polymer interface. More importantly, when ultrasonication was applied in the electroplating bath, the strong cavitation effect shook off the loosely-embedded nanosheets in the metal deposits, resulting in low incorporation content. Additionally, nanosheets with excessive positive charges could adhere to the cathode surface during electrodeposition, prohibiting further deposition and even terminating the co-deposition process. In contrast, nanosheets with a net negative charge are repelled away from the cathode surface and become entrapped in the nickel deposits instead of being incorporated into the metal deposits by electrophoresis [20], and thus diminishing the quality of deposits and reducing the nanosheets being incorporated into the metal deposits. Most dispersions of the nanosheets were studied in water. The authors are unware of any published study on the dispersion of MoS_2_/WS_2_ nanosheets in a divalent nickel sulphamate electrolyte solution. The particle size and zeta potential of these nanosheets should be studied in the electrolyte solution, which is critical for the composite mould fabrication via micro-electroforming. It is also important to note that the main drawbacks of ultrasonication include heating the bath and inducing defects in the nanosheets after excessive treatment times [18], [28]. Ultrasonic treatment time should be optimized to avoid overheating of the solution and a thermal sensor should be used to control the temperature of the electrolyte.

To date, there has been no research studying the scale-up co-deposition of MoS_2_/WS_2_ nanosheets and nickel for fabricating wafer-scale composite moulds. The reaggregation of MoS_2_/WS_2_ nanosheets in a nickel sulphamate solution has not been studied. The performance of a patterned four-inch composite mould remains unknown. Therefore, a primary objective of this study is to develop a new scale-up system for dispersing MoS_2_/WS_2_ nanosheets in the electrolyte solution. This has been achieved by using a dual-bath setup to achieve simultaneous dispersion and long-time deposition of 2D materials, where one bath is used to electrodeposit 2D materials, and the other is used to intermittently disperse the nanomaterials by means of ultrasound. The use of this dual-bath ensured effective dispersion during electrodeposition without any direct affect on the electrochemical reactions at the plating bath.

The amount of surfactants, ultrasonic power and time were all optimized to achieve uniform distribution of the nanosheets. The stability of the MoS_2_/WS_2_ nanosheets in the nickel sulphamate solution was evaluated to apply a second ultrasonication process to avoid the reaggregation of nanosheets during the electroforming process. The mechanism by which the surfactants and ultrasonic treatment dispersed the nanosheets was revealed in this study. The effects of the incorporated MoS_2_/WS_2_ nanosheets on the crystal structure, surface morphology, surface roughness and surface wettability of the composite moulds were studied systematically. The hardness enhancement and lubrication mechanism of the Ni-MoS_2_/WS_2_ composite moulds were revealed. Finally, micro hot embossing was performed on PMMA and COC 8007 components to evaluate the demoulding behaviour of the pure nickel mould and the Ni-MoS_2_/WS_2_ nanocomposite moulds.

## 2 Materials and methods

### 2.1 Experimental setup development

**Fig. 1:**
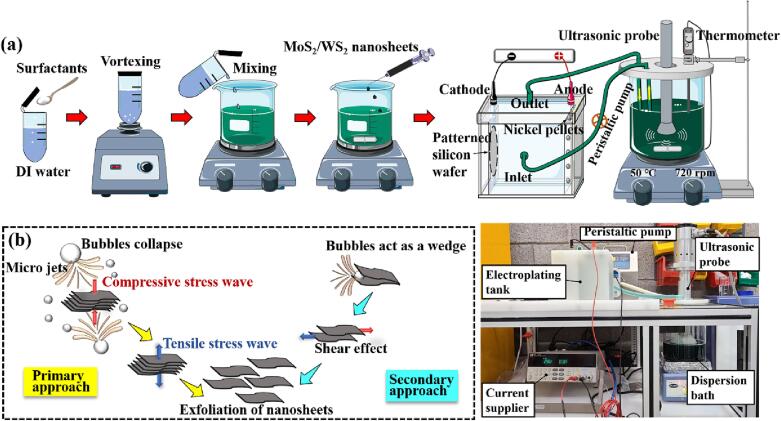
Preparation method for MoS_2_/WS_2_ nanosheets dispersion in the nickel sulphamate solution, the schematic diagram and picture of ultrasonic-assisted electroforming system of Ni-MoS_2_/WS_2_ nanocomposite moulds (a) and the dispersion mechanism by ultrasonication (b).

Fig. 1(a) illustrates the ultrasonic-assisted dual-bath electrodeposition setup that was developed for dispersing MoS_2_/WS_2_ nanosheets in the nickel sulphamate solution. This setup includes two baths. One bath aims to disperse the nanosheets in the electrolyte by an ultrasonic probe, which hangs over the bath to allow efficient ultrasonic cavitation; a PTFE-coated thermometer is immersed into the solution to detect the actual temperature, and a sensor is connected to the hot plate to adjust and maintain the temperature at 50 ± 3 °C and thus to avoid overheating the solution. When the detected temperature exceeded 50 °C, the hot plate shut off to avoid overheating. When the detected temperature was below 50 °C, the hot plate turned on and started heating the solution. Therefore, using this temperature probe, the temperature of the electrolyte solution can be maintained within 50 ± 3 °C. A cylindrical PTFE-coated steel magnetic stirrer is used on the bottom central area of the beaker for bath agitation. A peristaltic pump is used to pump the solution from the sonicated bath into the plating bath. The inlet of the plating tank is located in the centre of the sidewall, and the outlet is located at the top of the tank, ensuring that the plating tank is fully filled with the electrolyte solution.

The mechanism of dispersion of the nanosheets by means of ultrasonic treatment can be explained as follows. The primary way of dispersion is that the sonication leads to liquid cavitation, and the nanosheets are surrounded by these cavitation-induced bubbles (Fig. 1(b)). Once these bubbles collapse, micro-jets and shock waves then act on the surface of the nanosheets, leading to a compressive stress wave propagating throughout the nanosheets. Consequently, a tensile stress wave is reflected back to the nanosheet. As a result of the collapse of numerous bubbles, intense tensile stress waves form on these nanosheets, causing them to exfoliate [26]. The secondary way is that the micro-bubbles collapse and the resulting micro-jets act as a wedge inserted into the interlayer. The unbalanced lateral compressive stress causes a shear effect on the adjacent nanosheets, causing them to separate. Due to the high-aspect-ratio of these 2D nanosheets, more micro-jets act on the nanosheet surface than the nanosheet edge [27]. Therefore, it is the cavitation effect that is more dominant in exfoliating and dispersing these 2D nanosheets.

### 2.2 Preparation of electroforming electrolytes containing MoS_2_/WS_2_ nanosheets

A precise amount of surfactant was weighed carefully and dissolved in the deionised (DI) water with the use of a vortex mixer (Fig. 1(a)). Then, the surfactant solution was added to the nickel sulphamate solution. 1.0 mg/mL water-based MoS_2_/WS_2_ nanosheets (Jiangsu XFNano Materials Tech Co. Ltd., China) were injected slowly into the solution, drop by drop, to ensure they were fully charged by the surfactants. The mixed electroforming solution was then treated with a sonicator for 15 min. The compositions of the electroforming bath are listed in Table 1. Here, the cationic surfactant CTAB (Sigma–Aldrich, USA) (0.1 ∼ 1.0 g/L), anionic surfactant SDS (Sigma–Aldrich, USA) (0.1 ∼ 1.0 g/L), and the combination of CTAB and SDS (CTAB = 1.0 g/L, SDS (0.1 ∼ 1.0 g/L)) were used to detect the effect of surfactants on the particle size and surface charge of the MoS_2_/WS_2_ nanosheets in the nickel sulphamate solution.

**Table 1:** Electroforming bath compositions and process parameters for the fabrication of nickel/micro/nanocomposite moulds.

Nickel sulphamate (Ni((NH_2_SO_3_)_2_·4H_2_O)	∼400 g/L
Boric acid (H_3_BO_3_)	∼35 g/L
Wetting agent	1 mL/L
Tungsten disulfide (WS_2_)	0.1 g/L
Molybdenum disulfide (MoS_2_)	0.1 g/L
SDS	0.1 ∼ 1.0 g/L
CTAB	0.1 ∼ 1.0 g/L
PH	4.0
Temperature	50 °C
Current density	5 A/dm2
Plating time	3.5 h
Magnetic stirring speed	720 rpm

### 2.3 Characterization of MoS_2_/WS_2_ nanosheets in the nickel sulphamate solution

Hydrodynamic diameter and surface charge are two important parameters to evaluate the tendancy of the nanosheets to disperse in the solution. After the MoS_2_/WS_2_ nanosheets were fully dispersed with CTAB and/or SDS surfactants in the nickel sulphamate solution and treated with the sonication probe (Cheetsonic ultrasonics, power: 500 W, frequency: 20 KHz), the dispersibility of these nanosheets was examined by measuring the average particle size and mean zeta potential by particle analyser Litesizer 500 (Anton Paar). Each measurement was repeated three times and averaged. Meanwhile, the polydispersity index (PDI) was also measured from the zeta sizer for nanosheet distribution analysis. In this study, surfactants with different concentrations were used to investigate their effect on nanosheet dispersibility. After optimizing the amount of the surfactant, the formulation of the electroforming solution was fixed, and the prepared solution was treated by a sonicator with different ultrasonic power (amplitude 50 ∼ 100%) and time (0 ∼ 45 min). Once the ultrasonic power and time were also optimized, the treatment parameters were fixed. MoS_2_/WS_2_ nanosheets are prone to aggregate in solution. Therefore, after ultrasonic treatment, the influence of settling time on the particle size was investigated to determine the time at which the sonication treatment for the second time should be started so as to avoid aggregation of these nanosheets in the electrolyte solution.

### 2.4 Fabrication of Ni-MoS_2_/WS_2_ nanocomposite moulds by UV-LIGA.

The Ni-MoS_2_/WS_2_ nanocomposite moulds were electroformed by an ultrasonic-assisted dual-bath plating system (Fig. 1(a)). A micro-structured 4-inch silicon wafer was held inside the tank and served as a cathode. A titanium basket containing nickel pellets served as an anode. The gap distance between the cathode and the anode was maintained at ∼6 cm. The titanium basket and nickel pellets were washed with isopropanol and DI water and activated with sulphamic acid before electroforming.

**Fig. 2:**
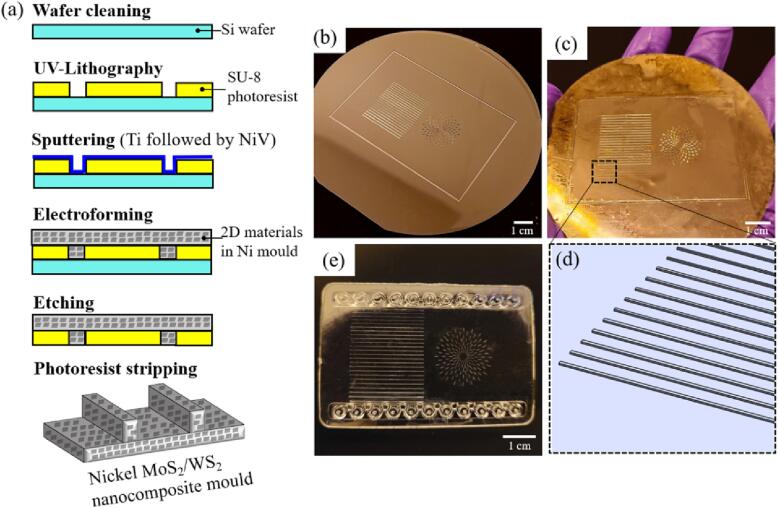
Schematic of UV-LIGA process to fabricate micro structured Ni-MoS_2_/WS_2_ nanocomposite moulds (a); the patterned silicon wafer fabricated from UV-lithography (b); the electroformed Ni/MoS_2_ nanocomposite mould (c); the feature design of micro ridges on the mould with a width of 100 µm and a height of 100 µm (d) and the hot-embossed PMMA chip (e).

To fabricate the silicon wafer with micro-features, the UV-LIGA process was performed (Fig. 2(a)). Before UV-lithography, the silicon wafer was washed with acetone, isopropanol, and DI water for 10 min by ultrasonic bath, followed by drying on the hot plate at 120 °C for 1 h and plasma treatment for 7 min to remove any contamination. The SU-8 photoresist (GM 1075, Gersteltec Sarl) was spin-coated on the silicon wafer, followed by the pre-bake and the exposure of the pattern with a UV-LED masking system (UV-KUB 2, Kole). After this, post-bake was performed. The silicon wafer with photoresist was immersed in the PGMEA (99.5%, Sigma Aldrich) to wash away any uncrosslinked photoresist, and the micro-features on the silicon wafer surface were developed in this step. Isopropanol (98%, Sigma-Aldrich) was used finally to rinse off the residual PGMEA. The patterned silicon wafer is shown in Fig. 2(b). After UV-lithography, the metallisation of the patterned silicon wafer was achieved by sputtering 50 nm titanium and 200 nm nickel vanadium on its surface.

Before electroforming, the surfactants with optimized amounts were dissolved in the DI water and then added to the nickel sulphamate solution. MoS_2_/WS_2_ nanosheets were dispersed in the electrolyte solution using the optimized ultrasonic dispersion parameters. The bath parameters are noted in Table 1. After electroforming, a nickel composite mould with a thickness of ∼ 500 nm was obtained (Fig. 2(c)). A pure nickel mould was fabricated with the same UV-LIGA process. The micro ridges had a width of 100 µm and a height of 100 µm and were selected to study the effects of demoulding from both the Ni-MoS_2_/WS_2_ composite moulds and the pure nickel mould (Fig. 2(d)). Then, the silicon wafer was etched with 30% KOH solution at 80 °C. The photoresist was removed (Strip A, Gersteltec Sarl) at 150 °C for 1 h to expose the mirror-like working surface of the Ni-MoS_2_/WS_2_ nanocomposite mould for characterisation.

### 2.5 Characterisation of Ni-MoS_2_/WS_2_ nanocomposite moulds

To expose the MoS_2_/WS_2_ nanosheets on the nickel nanocomposite moulds surfaces, a small blank area (1 cm × 1 cm) was selected on the mould surface and etched with Marble's reagent for 10 s. After etching, the surface morphologies of the pure nickel mould and composite moulds were imaged by scanning electron microscopy (SEM, Hitachi Topdesk 4000). The content of MoS_2_/WS_2_ nanosheets incorporated into the composite mould was quantified by the energy dispersive X-ray analysis (EDS). The phase compositions of all moulds were detected by Renishaw Raman spectrometer with a laser wavelength of 785 nm. The phase structures and crystallite size of the pure nickel mould and composite moulds were analysed using an X-ray diffractometer (Siemens D500), scanned at 0.04°/s in the two-theta range from 10° to 90°. The microhardness of the pure nickel mould and composite moulds was measured on the cross-section using a Vickers hardness tester (Buehler Micromet-4). The load was 50 g applied for 15 s in eight different positions, and the microhardness value was averaged. The mould surface roughness was measured by an optical 3D profilometer (NPFlex).

Water contact angles (WCA) were measured with a contact angle goniometer (Ossila) five times with a water volume of 10 µL for each test. PMMA (ALTUGLAS VSUVT) and COC 8007 (TOPAS) melt contact angles (CA) were also measured on the surfaces of the pure nickel mould and the Ni-MoS_2_/WS_2_ composite moulds using a drop shape analyser (Krüss DAS100E) to identify the mould surface hydrophobicity. During the polymer melt CA test, the mould was placed in the heating chamber with a solid polymer pellet on top of its surface. The mould-polymer system was heated to above the melting temperature of the polymer, and the CA was measured until the polymer was a fully melted drop. Here, the melt temperatures of the PMMA and COC 8007 were ∼195 °C and ∼204 °C respectively, and the test temperature was set at ∼205 °C (for PMMA) and ∼215 °C (for COC 8007). The test temperature was the processing temperature of these polymers for micro injection moulding of microfluidic chips. The volume of the polymer melt was set as ∼5 µL. Each CA was measured three times to ensure repeatability.

The friction and wear behaviour of the pure nickel mould and composite moulds were assessed using a pin-on-disk tribometer (ISC200PC). A PMMA (ALTUGLAS VSUVT) pin with a diameter of 2 mm and a COC 8007 (TOPAS) pin with a diameter of 3 mm were used for the friction tests with a normal contact load of 1 N and a rotating speed of 31.8 rpm for 10 min. After the pin-on-disc test, to evaluate the mould wear resistance to the polymer material, the wear morphology and surface roughness of the mould were imaged by SEM and measured by a 3D profilometer, respectively. The pin-on-disc tests were conducted under unlubricated conditions at room temperature and ambient air.

### 2.6 Validation of lubricating properties of the Ni-MoS_2_/WS_2_ nanocomposite moulds

To compare the demoulding effects of the pure nickel mould and nickel-MoS_2_/WS_2_ nanocomposite moulds, micro hot embossing was performed with injection-moulded plain PMMA (ALTUGLAS VS-UVT) chips and COC 8007 (TOPAS) chips (Fig. 2(e)). During micro hot embossing, these polymer chips were capable of replicating the micro features from the moulds. The parameters of micro hot embossing are listed in Table 2. The glass transition temperature (Tg) of PMMA was ∼99.7 °C, and the hold temperature was set as 120.0 °C while a load of 2500 N was applied for 5 min. The demoulding temperature was set as 90 °C for PMMA. The Tg of COC 8007 was ∼78 °C, and the hold temperature and pressure of 95 °C and 9000 N were applied for 5 min. The demoulding temperature of COC 8007 was 70 °C. The demoulding temperature was set below the Tg of the polymer to ensure complete solidification of micro features on the chip.

**Table 2:** Micro hot embossing parameters of PMMA and COC 8007 chips.

Materials	Glass transition temperature (Tg) (℃)	Hold temperature (℃)	Hold pressure (N)	Hold time(min)	Demoulding temperature (℃)
PMMA	∼99.7	120.0	2500	5	90
COC 8007	∼78.0	95.0	9000	5	70

## 3 Results and discussion

### 3.1 Characterization of MoS_2_/WS_2_ nanosheets

**Fig. 3:**
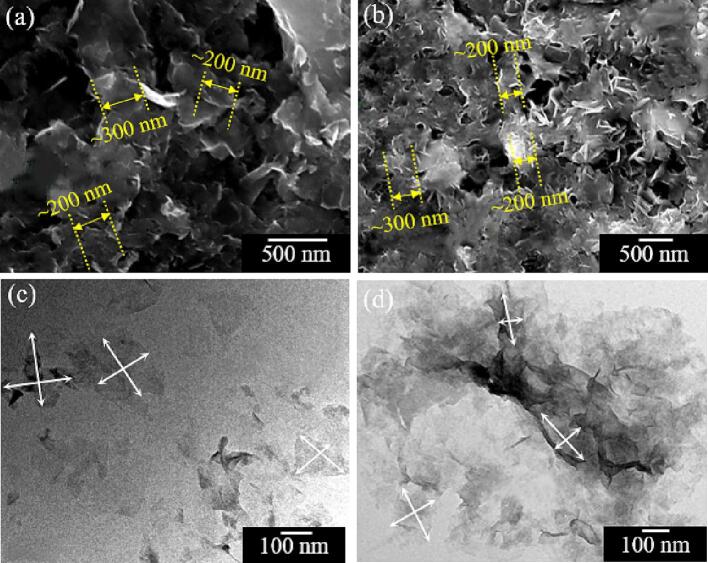
SEM images of (a) MoS_2_ and (b) WS_2_ nanosheets; and TEM images of (c) MoS_2_ and (d) WS_2_ nanosheets.

Fig. 3 shows the morphology of the MoS_2_/WS_2_ nanosheets characterized by SEM and TEM, respectively. The SEM results (Fig. 3(a and b)) showed that MoS_2_/WS_2_ nanosheets were nearly monodisperse with a width ranging from 200 nm to 300 nm. Both MoS_2_ and WS_2_ nanosheets were stacked loosely. The TEM results indicated the thin layered structures with the dimension distributions of most nanosheets in the range of 200 nm to 300 nm, which was in accordance with the morphology in the SEM images. MoS_2_/WS_2_ samples were mainly comprised of ultrathin and well-dispersed sheets. It was noticed that some MoS_2_/WS_2_ nanosheets were slightly curved with small wrinkles, as had also been found by others [11], [28]. This could be due to the instability of 2D materials. To eliminate the dangling bonds at the edges, these nanosheets were prone to roll up to form closed structures, as also proved by related literatures [29], [30]. These layered nanosheets are expected to provide the lubricating properties of the Ni-MoS_2_/WS_2_ nanocomposite moulds.

### 3.2 Characterization of MoS_2_/WS_2_ nanosheets in the electrolyte solution

#### 3.2.1 Optimization of surfactants

Surfactants play two roles in dispersing MoS_2_/WS_2_ nanosheets in the nickel sulphamate solution: one is adjusting the surface tension of the solution, increasing the solubility of the nanosheets; the other is charging the nanosheets to achieve a stable dispersion by electrostatic repulsion [8]. Here, pure CTAB, pure SDS and the mixture of 1.0 g/L CTAB and SDS were applied to charge and disperse MoS_2_/WS_2_ nanosheets. MoS_2_/WS_2_ nanosheets are charge-free, and their net surface charge is determined by the surfactants added to the solution, as proved by Gupta et al. [31]. Cationic surfactant CTAB gives positive charges, and anionic surfactant SDS offers negative charges to these nanosheets, and the nanosheets are modified by these surfactants to show either net positive or net negative charges, similar to what was studied in previous research [14].

**Fig. 4:**
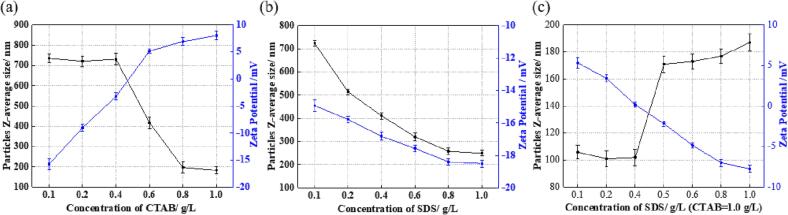
The effect of concentrations of different surfactants on the particle size and zeta potential of MoS_2_ nanosheets in the nickel sulphamate solution: (a) CTAB; (b) SDS; and (c) SDS (CTAB = 1.0 g/L).

**Fig. 5:**
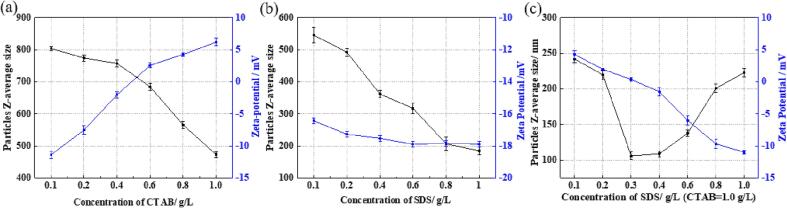
The effect of concentrations of different surfactants on the particle size and zeta potential of WS_2_ nanosheets in the nickel sulphamate solution: (a) CTAB; (b) SDS; and (c) SDS (CTAB = 1.0 g/L).

To evaluate the dispersibility of MoS_2_/WS_2_ nanosheets in the nickel sulphamate solution, the particle Z-average size and zeta potential were measured by the Dynamic Laser Scattering particle analyser Litesizer. Fig. 4, Fig. 5 show the relationship between MoS_2_/WS_2_ nanosheet size, zeta potential and different concentrations of surfactants. According to Fig. 4(a), with an increase of CTAB concentration from 0.1 g/L to 1.0 g/L, the size of MoS_2_ nanosheets decreased from ∼734 nm to ∼184 nm, while the zeta potential increased from −15 mV to 8 mV. Similarly, the size of WS_2_ nanosheets decreased from ∼804 nm to ∼473 nm, while the zeta potential increased from −11 mV to 6 mV (Fig. 5(a)). It was noticed that when the CTAB concentration was higher than 0.4 g/L, the net surface charge of MoS_2_ nanosheets changed from negative to positive, and there was a significant decrease in the MoS_2_ nanosheet size (from 730 nm at 0.4 g/L CTAB to 418 nm at 0.6 g/L CTAB). This might be because low concentrations of CTAB cannot be strongly bound to the MoS_2_ nanosheets, and most CTAB is free surfactant molecules in the electrolyte solution, similar to others [31]. When CTAB concentration exceeded 0.6 g/L, the amount of bond surfactants was higher than that of free surfactants, resulting in a stable positive charge repulsion and decreased nanosheet size. A similar result was found in the WS_2_ nanosheets (Fig. 5(a)). When CTAB concentration exceeded 0.4 g/L, the zeta potential shifted from −2.0 mV to 2 mV, with the decrease of nanosheet size from ∼760 nm to ∼684 nm. The increase of CTAB concentration (higher than 0.6 g/L) resulted in a sharp decrease in nanosheet size in both MoS_2_ and WS_2_ nanosheets, indicating that a high concentration of CTAB is a better option for dispersing the MoS_2_/WS_2_ nanosheets to form stable positive charge repulsion.

With the increasing addition of SDS, the size of MoS_2_ nanosheets gradually reduced from ∼723 nm to ∼258 nm, with the zeta potential decreasing slightly from −15 mV to −18 mV. Similarly, the size of WS_2_ nanosheets decreased from ∼545 nm at 0.1 g/L SDS to ∼184 nm at 1.0 g/L SDS. The zeta potential decreased in a small range from −16 mV to −18 mV.

The most efficient dispersion of both MoS_2_ and WS_2_ nanosheets was achieved by using cationic-rich surfactant mixtures, represented as the smallest nanosheet size achieved under the synergistic effects of both cationic and anionic surfactants (Fig. 4(c) and Fig. 5(c)). The combined use of CTAB and SDS reduced the size of MoS_2_ nanosheets from ∼106 nm to ∼102 nm at 1.0 g/L CTAB and 0.4 g/L SDS. The further addition of SDS did not facilitate the reduction of nanosheet size, represented as the further increase of the nanosheet size to ∼187 nm at 1.0 g/L CTAB and 1.0 g/L SDS. The zeta potential was 0.16 mV at 1.0 g/L CTAB and 0.4 g/L SDS, and this value became more negative with the increase of SDS concentration. When 1.0 g/L CTAB and 1.0 g/L SDS were added to the solution, MoS_2_ nanosheets presented a negative surface charge with a zeta potential of −7.74 mV. This is because the SDS molecules have a shorter tail than CTAB, resulting in easier adsorption to the nanosheets surface and greater packing, thus more negative charges at the same concentration of CTAB and SDS. Similar results were obtained by others [32], [33]. As the case for WS_2_ nanosheets, the smallest nanosheet size of ∼ 106 nm was achieved at 1.0 g/L CTAB and 0.3 g/L SDS, with a net positive surface charge of 0.38 mV. With the further addition of SDS, the nanosheet size increased to ∼223 nm, and the zeta potential became more negative to −10.98 mV.

Besides the particle size and zeta potential, the polydispersity index (PDI) value of the nanosheet distribution is also an important parameter to indicate the aggregation of nanosheets [34]. PDI represents the average uniformity of particles in the solution and ranges from 0 to 1. A PDI value of 0 indicates a monodisperse solution with a perfectly uniform distribution of particles. A PDI value above 0.4 suggests a very broad particle size distribution and indicates aggregation of some particles in the solution [35]. Table 3, Table 4 show the PDI of MoS_2_ and WS_2_ nanosheets modified with different surfactants in the electrolyte solution. All nanosheets had a PDI below 0.4, regarded as “moderate polydispersity” [36]. Particularly, the combined use of CTAB and SDS can effectively reduce the PDI from 0.2 ∼ 0.3 for pure surfactants to lower than 0.2 for MoS_2_ nanosheets, indicating a uniform particle-size distribution of MoS_2_ nanosheets in the nickel sulphamate solution (Table 3). The PDI of WS_2_ nanosheets dispersed by the mixture surfactants was also very narrow (∼0.2), showing the synergistic effect of mixed surfactants in dispersing these hydrophobic nanosheets in the electrolyte solution (Table 4).

**Table 3:** The polydispersity index (PDI) of MoS_2_ nanosheets in the nickel sulphamate solution dispersed with different surfactants.

CTAB (g/L)	PDI	SDS (g/L)	PDI	CTAB (1.0 g/L) + SDS (g/L)	PDI
0.1	0.24	0.1	0.27	0.1	0.18
0.2	0.25	0.2	0.23	0.2	0.15
0.4	0.23	0.4	0.21	0.4	0.14
0.6	0.27	0.6	0.2	0.5	0.15
0.8	0.23	0.8	0.19	0.6	0.18
1.0	0.22	1.0	0.13	0.8	0.16
				1	0.15

**Table 4:** The polydispersity index (PDI) of WS_2_ nanosheets in the nickel sulphamate solution dispersed with different surfactants.

CTAB (g/L)	PDI	SDS (g/L)	PDI	CTAB (1.0 g/L) + SDS (g/L)	PDI
0.1	0.17	0.1	0.29	0.1	0.19
0.2	0.14	0.2	0.23	0.2	0.15
0.4	0.19	0.4	0.27	0.4	0.17
0.6	0.24	0.6	0.29	0.5	0.21
0.8	0.21	0.8	0.28	0.6	0.18
1.0	0.19	1.0	0.26	0.8	0.23
				1	0.15

**Fig. 6:**
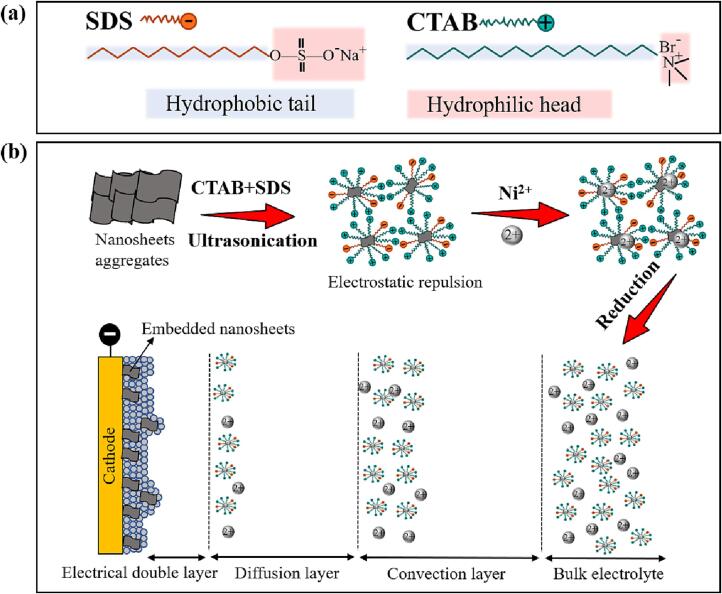
The chemical structures of surfactants SDS and CTAB (a); the dispersion mechanism of MoS_2_/WS_2_ nanosheets in the nickel sulphamate solution by surfactants CTAB and SDS under ultrasonication and the electrodeposition process of Ni-MoS_2_/WS_2_ nanocomposite moulds.

It should be noted that a net negative charge on the MoS_2_/WS_2_ is not favourable for the electrodeposition of these nanosheets to the cathode. During the co-deposition of nickel and MoS_2_/WS_2_ nanosheets, the migration of these nanosheets towards the cathode surface is governed by electrophoresis and convective-diffusion mass transportation. The former is the migration of charged nanosheets under the action of an electric field. The latter is the movement driven by the velocity gradients in the convection layer and the concentration gradients in the diffusion layer. The steps are illustrated in Fig. 6 and summarised as follows: (i) nanosheets are charged by surfactants adsorbed on their surfaces, (ii) physical transportation of nanosheets through the convection layer, (iii) mass transportation of nanosheets through the diffusion layer, (iv) migration of nanosheets driven by the potential gradient through the electrical double layer, (v) nanosheets are physically embedded into the nickel deposit along with the reduction of nickel ions [37]. Here, zeta potential plays two critical roles: stabilising the dispersion of these charged nanosheets via a certain degree of electrostatic repulsion and determining the migration direction of charged nanosheets. Therefore, the nanosheets need to acquire a net positive charge to facilitate their migration from the bulk electrolyte to the cathode surface. Consequently, 1.0 g/L CTAB with 0.4 g/L SDS was selected for dispersing MoS_2_ nanosheets, and 1.0 g/L CTAB with 0.3 g/L SDS was used for the dispersion of WS_2_ nanosheets in the nickel sulphamate solution to achieve good dispersion and a net positive charge on the nanosheet surfaces. Additionally, the net positive charge should be maintained at a proper level. Otherwise, too many positive charges can cause aggressive deposition of 2D materials with porous structures, reducing the deposit quality. This would further prevent nickel deposition and stop the formation of the dense and compact structures of the composite mould.

#### 3.2.2 Optimization of ultrasonic power and time

**Fig. 7:**
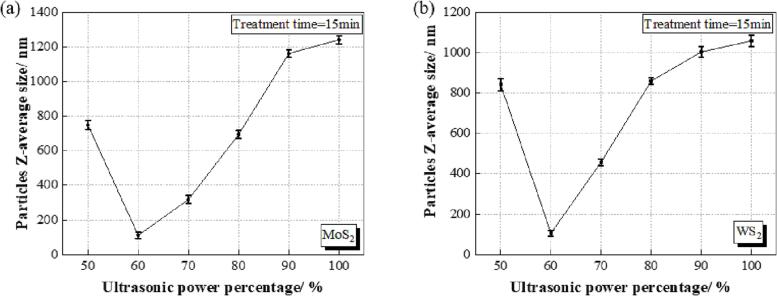
The effect of ultrasonic power on the particle size of (a) MoS_2_ and (b) WS_2_ nanosheets in the nickel sulphamate solution.

Fig. 7 shows the effect of ultrasonic power on the nanosheet size of MoS_2_ and WS_2_. The treatment time was set as 15 min for each treatment. With the increase of ultrasonic power percentage from 50% to 100%, the size of MoS_2_ nanosheet decreased from ∼748 nm at 50% to ∼110 nm at 60%, and further increase of the ultrasonic power induced the aggregation of MoS_2_ nanosheets, represented as the nanosheet size increasing gradually to ∼1240 nm (Fig. 7(a)).

A similar result was also found in WS_2_ nanosheets (Fig. 7(b)). The smallest nanosheet size of ∼106 nm was achieved at 60% ultrasonic power, and then the sheet size increased sharply to ∼1058 nm at 100% power, indicating WS_2_ nanosheets aggregated in the solution. Additionally, the PDI of MoS_2_ and WS_2_ nanosheets achieved the smallest value (0.22 for MoS_2_ and 0.20 for WS_2_) when treated with 60% ultrasonic power, indicating a uniform distribution of these nanosheets in the nickel sulphamate solution without nanosheet aggregates (Table 5) [36].

**Table 5:** The polydispersity index (PDI) of MoS_2_ and WS_2_ nanosheets in the nickel sulphamate solution treated with different ultrasonic power for 15 min.

Ultrasonic power (%)	PDI (MoS_2_)	PDI (WS_2_)
50	0.27	0.31
60	0.22	0.20
70	0.28	0.33
80	0.33	0.27
90	0.35	0.31
100	0.34	0.33

Moderate ultrasonic power is more effective in dispersing these nanosheets, as also found by Tyurnina et al. [38]. This could be explained by the ultrasonic cavitation effect. For a liquid with fixed composition that is subjected to ultrasound, the cavitation intensity rises with the increasing input power of the ultrasound. This cavitation effect tends to peak once the ultrasonic intensity reaches a certain level. In the present study, a power level of 50% was not sufficient to break up MoS_2_ and WS_2_ nanosheets, while a 60% input power generated stable cavitation that served to exfoliate and disperse these nanosheets. As the input power increased further, the cavitation intensity decreased: input power levels ranging from 70% to 100% generated increasingly larger nanosheets. This was due to the ultrasonic cavitation shielding effect. The increased number of bubbles beneath the sonication probe hindered the transmission of acoustic waves and the generation of inertial cavitation, which, in turn, led to less effective exfoliation of the nanosheets. This was also shown by Qiao et al. [39] and Sutkar et al. [40]. In this present study, many vain bubbles were produced at input power levels ranging from 70% to 100%. Larger levels of input power led to greater numbers of vain bubbles being generated; these bubbles increased the scattering attenuation and decreased the cavitation intensity. Consequently, 60% power is appropriate for dispersing both the MoS_2_ and WS_2_ nanosheets in the nickel sulphamate solution and thus to achieve the smallest nanosheet size.

**Fig. 8:**
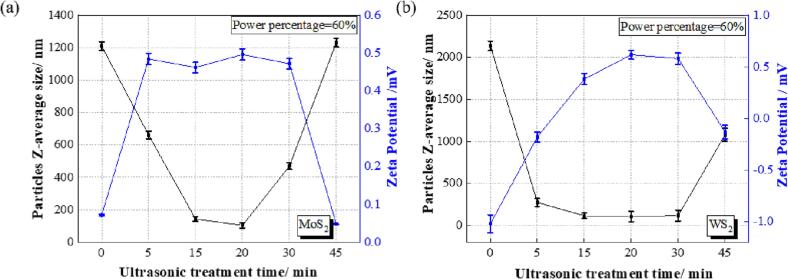
The effect of ultrasonic treatment time on the particle size and zeta potential of (a) MoS_2_ and (b) WS_2_ nanosheets in the nickel sulphamate solution.

Fig. 8 shows the effect of ultrasonic treatment time on the size and zeta potential of MoS_2_ and WS_2_ nanosheets. The untreated MoS_2_ nanosheets had a size of ∼1209 nm with a zeta potential of 0.07 mV. With increasing treatment time, there was a steep decline in the MoS_2_ nanosheet size from ∼1209 nm to ∼661 nm after treatment for only 5 min, indicating that ultrasonic treatment is an effective method for nanosheet dispersion. The minimum sheet size of ∼104 nm was achieved after 20 min, with a positive zeta potential of 0.49 mV (Fig. 8(a)). The zeta potential value was stable when treated for 5 to 30 min, fluctuating between 0.46 and 0.50 mV. However, when treated for 45 min, the MoS_2_ nanosheets aggregated, represented by the nanosheet size of ∼1231 nm and zeta potential of 0.04 mV.

Similarly, the untreated WS_2_ nanosheets had an average size of ∼2134 nm. The WS_2_ nanosheet size was significantly reduced from ∼2134 nm to ∼274 nm when treated for only 5 min. A minimum sheet size of ∼106 nm was obtained, with a positive zeta potential of 0.62 mV (Fig. 8(b)). 30 min of ultrasonic treatment was still effective in dispersing WS_2_ nanosheets, providing an average sheet size of ∼117 nm and positive zeta potential of 0.58 mV. Long treating times, 45 min, caused WS_2_ nanosheets to agglomerate, having an average sheet size of ∼1076 nm and a zeta potential of −0.13 mV. Besides, as shown in Table 6, the PDI of MoS_2_/WS_2_ nanosheets was small (0.21 and 0.22), suggesting a narrow distribution of small particle sizes and a homogenous distribution of these nanosheets in the electrolyte solution without aggregation [36].

**Table 6:** The polydispersity index (PDI) of MoS_2_ and WS_2_ nanosheets in the nickel sulphamate solution treated with 60% ultrasonic power for different treatment times.

Ultrasonic treatment time (min)	PDI (MoS_2_)	PDI (WS_2_)
0	0.24	0.26
5	0.37	0.28
15	0.22	0.20
20	0.21	0.22
30	0.25	0.24
45	0.28	0.29

In conclusion, using an ultrasonic treatment with a power of 60% was suitable for dispersing MoS_2_/WS_2_ nanosheets for 20 min and thus achieved effective nanosheet dispersion. The average nanosheet size of ∼104 nm for MoS_2_ and ∼106 nm for WS_2_ was even smaller than the size indicated by the manufacturer (200 ∼ 300 nm), which indicated that a proper ultrasonic treatment that takes advantage of the synergistic effect of surfactants was useful in reducing the agglomerated sizes of nanosheets; this has also been reported elsewhere [9], [24], [32]. Longer ultrasonic treatment times would lead to more agglomeration of nanosheets and a decrease in exfoliation efficiency, as excessive ultrasonic treatment would cause a rapid increase in the local temperature of the reaction media, which could affect the nanosheet size and even produce nanosheets with topographical defects that would cause bulk disorder, as discussed by Bracamonte et al. [41] and Mehrali et al. [42]. An optimum sonication time could be achieved for exfoliating nanosheets, after which the aggregation rate becomes more pronounced than the exfoliation rate, leading to a decrease in the exfoliation effect [43]. Research by Hadi et al. [43] also concluded that longer sonication times cause the exfoliation efficiency to decline, probably due to oxidative degradation of the solvent.

#### 3.2.3 MoS_2_/WS_2_ stability in the nickel sulphamate solution

60% ultrasonic power was selected as being optimum for treating MoS_2_/WS_2_ nanosheets for 20 min before electrodeposition in order to achieve effective nanosheet dispersion. A uniform composition of the electrolyte solution is critical to producing the Ni-MoS_2_/WS_2_ nanocomposite moulds, which requires sufficient agitation in the device for dispersing these nanosheets. Without sufficient dispersion, nanosheet aggregates first develop in the electrolyte solution and then adhere to the cathode or anode surface, depending on their net surface charge. Once the cathode or anode is covered with these hydrophobic nanosheets, the electrodeposition efficiency will be largely reduced. The co-deposition of nanosheet aggregates can result in roughened, dendritic structures or powdery deposits instead of compact structures, especially at high current density, as found in our previous study [14] and also by others [20].

**Fig. 9:**
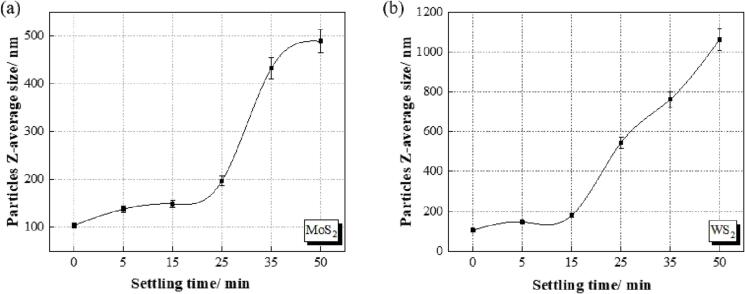
The particle size of (a) MoS_2_ and (b) WS_2_ nanosheets as a function of settling time after ultrasonic treatment.

During the co-deposition of MoS_2_/WS_2_ nanosheets and nickel, these nanosheets were still prone to sedimentation, and precipitation, finally developing into aggregates. Agglomeration of these nanosheets was found in the electrolyte solution, even applied with magnetic stirring and pre-treated with an ultrasonic probe and with the assistance of surfactants, as also found in other research [13]. Therefore, the change of nanosheet size was detected with the settling time after the ultrasonic treatment (Fig. 9). During settling, a magnetic stirrer was used with a stirring speed of 720 rpm, which was compatible with our electroplating setup.

Immediately after the treatment, MoS_2_ nanosheets and WS_2_ nanosheets had sizes of ∼104 nm and ∼106 nm respectively. In the first 25 min of magnetic stirring, the MoS_2_ nanosheets had uniform sizes of ∼138 nm after 5 min, ∼148 nm after 15 min and ∼197 nm after 25 min, respectively, indicating a stable dispersion effect (Fig. 9(a)). However, large nanosheet sizes were detected as this agitation was continued for longer times (∼432 nm at 35 min and ∼488 nm at 50 min). As for the WS_2_ nanosheets, uniform dispersion was maintained for the first 15 min with a sheet size of ∼145 nm at 5 min and ∼179 nm at 15 min. After 15 min, WS_2_ nanosheets agglomerated, as evidenced by the sheet sizes increasing to ∼544 nm after 25 min, ∼762 nm after 35 min and ∼1062 nm after 50 min (Fig. 9(b)).

As shown in Table 7, the PDI remained below 0.3 for MoS_2_ nanosheets for all settling times, while this value went up to 0.37 for WS_2_ nanosheets when the settling time was 50 min after the ultrasonic treatment: this indicated that the MoS_2_ had moderate nanosheet sizes (∼500 nm) with a narrow particle distribution (PDI of 0.27 after 50 min), while WS_2_ had both large nanosheet sizes (∼1062 nm after 50 min) and broad particle size distribution (PDI of 0.37 after 50 min), which necessitated a second ultrasonic treatment to achieve better dispersion. Therefore, to avoid agglomeration of nanosheets during electroforming, ultrasonic treatment should be applied regularly before these nanosheets develop into aggregates. Consequently, ultrasonic treatment was applied every 25 min for MoS_2_ and every 15 min for WS_2_ during the co-deposition process.

**Table 7:** The polydispersity index (PDI) of MoS_2_ and WS_2_ nanosheets in the nickel sulphamate solution as a function of settling time after ultrasonic treatment.

Settling time (min)	PDI (MoS_2_)	PDI (WS_2_)
0	0.24	0.2
5	0.25	0.25
15	0.21	0.26
25	0.21	0.25
35	0.24	0.28
50	0.27	0.37

After surfactant amount and ultrasonic power and time were optimized, 1.0 g/L CTAB with 0.4 g/L SDS was used to charge MoS_2_ nanosheets while 1.0 g/L CTAB with 0.3 g/L SDS was used for WS_2_ nanosheets in the nickel sulphamate solution. Both solutions were treated with an ultrasonic probe with a power percentage of 60% for 20 min before electroforming. After every 25 min and every 15 min, ultrasonic treatment was applied regularly to ensure dispersion of the MoS_2_ and WS_2_, respectively.

### 3.3 Characterization of Ni-MoS_2_/WS_2_ nanocomposite moulds

#### 3.3.1 Surface morphology and compositions analysis

**Fig. 10:**
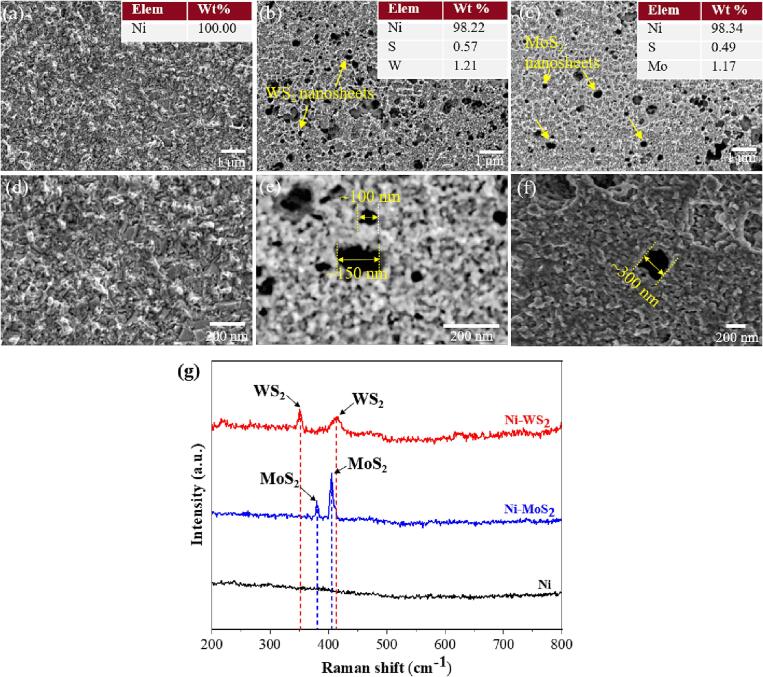
Surface morphology and the weight percentages of elements based on the EDS analysis of pure nickel mould (a) and Ni/MoS_2_ mould (b) and Ni/WS_2_ mould (c) and their surface morphology of higher resolution (d ∼ f), (g) Raman spectrum of pure nickel mould and nickel-MoS_2_/WS_2_ nanocomposite moulds.

Fig. 10 shows the surface morphology of the pure nickel and nickel-MoS_2_/WS_2_ nanocomposite moulds at low and high resolutions. The element weight percentage of nickel and MoS_2_/WS_2_ nanosheets was characterized by EDS (Fig. 10(a ∼ c)). All mould sample surfaces were etched before SEM to expose the embedded MoS_2_/WS_2_ nanosheets. To further determine the compositions of all moulds and whether oxides were formed during electrodeposition, the Raman spectra were also considered, as shown in Fig. 10(g).

The pure nickel mould had a smooth and compact surface, as shown in Fig. 10(a and d), which consisted of 100 wt% nickel. The SEM result proved the successful dispersion and incorporation of MoS_2_/WS_2_ nanosheets into the nickel composite moulds. The exfoliation of MoS_2_ and WS_2_ nanosheets was also investigated using SEM by Arefi-Oskoui et al. [44]. In the present study, MoS_2_ nanosheets were found to be evenly distributed in the composite mould surface, with a molybdenum content of 1.17 wt%. Irregular WS_2_ nanosheets were distributed uniformly in the Ni/WS_2_ nanocomposite mould, with a tungsten content of 1.21 wt%. The incorporated WS_2_ nanosheets had smaller sizes (from ∼100 nm to ∼200 nm) than the MoS_2_ nanosheets (∼300 nm), as shown in Fig. 10(e and f). This is probably because the ultrasonic treatment was more frequently applied for dispersing WS_2_ (every 15 min) than MoS_2_ (every 25 min), resulting in smaller nanosheet sizes and a better dispersion effect.

The Raman spectra revealed that for composite moulds, peak 350 and peak 416 were detected in the Ni-WS_2_ nanocomposite mould, indicating the main phase of WS_2_ [45]. Peak 379 and peak 405 were determined in Ni-MoS_2_ nanocomposite mould, which was the main phase of MoS_2_ [46]. No phase of oxides of either WO_3_ or MoO_3_ was detected, indicating that the WS_2_ and MoS_2_ nanosheets remained intact in the nickel nanocomposite moulds after co-deposition. In addition, the absence of WO_3_ or MoO_3_ would suggest good tribological performance, since the presence of these oxides often leads to a degradation in the lubrication performance of metal composites [45].

During the co-deposition of nickel and MoS_2_/WS_2_ nanosheets, surfactants and nickel atoms adsorbed onto the nanosheet surfaces in the electrolyte solution. The adsorption of these surfactants to the nanosheets was a dynamic process. In a system consisting of surfactants CTAB, SDS and MoS_2_/WS_2_ nanosheets, a dynamic equilibrium exists between free surfactants in the electrolyte solution, surfactants bonded to the surfaces of the nanosheets and surfactants at the liquid–air interface [47]. The cationic surfactant CTAB provided positive charges, and the anionic surfactant SDS offered negative charges [31], [48]. The cationic-rich surfactants ensured there was a net positive charge on the nanosheet surfaces [47]. Here, both MoS_2_ and WS_2_ nanosheets had net positive surface charges, as shown previously in Section 3.2.2, ensuring that they were attracted to the cathode surface by electrophoresis and led to adsorption instead of being physically incorporated with the nickel deposits [14], [20], [48]. This is because, for a nickel composite mould with a compact structure and high mechanical strength, nanosheets must be attracted and stick to the cathode surface rather than glancing off the cathode surface due to the lack of incorporation into the growing nickel deposits coupled with excess momentum or high shear from the solution being mixed [20]. The ultrasonic-assisted circulating plating ensured the dispersion of these nanosheets before they developed into aggregates in one tank and the stable co-deposition process in the other tank, resulting in a uniform distribution of MoS_2_/WS_2_ nanosheets in the nickel composite moulds.

#### 3.3.2 XRD analysis

**Fig. 11:**
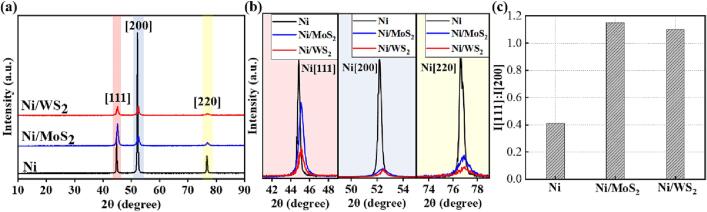
XRD spectra of (a) pure nickel mould, Ni/MoS_2_ and Ni/WS_2_ nanocomposite moulds; the detailed diffraction of (b) Ni[1 1 1], Ni[2 0 0] and Ni[2 2 0] of pure nickel mould, Ni/MoS_2_ and Ni/WS_2_ nanocomposite moulds; (c) peak intensity ratio of the Ni [1 1 1] to Ni[2 0 0] deduced from the spectra in (a).

Fig. 11 shows the XRD spectra of pure nickel and Ni-MoS_2_/WS_2_ nanocomposite moulds and the detailed diffraction of peaks [1 1 1], [2 0 0] and [2 2 0]. All nickel moulds had face-centred-cubic (FCC) structures, indicating that no chemical reaction occured between the Ni and MoS_2_/WS_2_ nanosheets during co-deposition [10]. For FCC metals, peak [1 1 1] has three slip planes while [2 0 0] has one slip plane. Therefore, [2 0 0] had less ability to deform during the microhardness indentation test, thus indicating higher microhardness [49], [50]. Of all the surface textures, [1 1 1] had the lowest surface energy, similar to that reported elsewhere [51], [52]. Therefore, the increased intensity of peak [1 1 1] corresponds to a decrease of the surface energy. As shown in Fig. 11(c), the peak intensity ratio of [1 1 1] to [2 0 0] was 0.41 for the pure nickel mould, while this ratio increased to 1.15 for Ni/MoS_2_ and to 1.10 for Ni/WS_2_ composites. This indicated the shift of preferred crystal orientation from [2 0 0] for pure nickel to [1 1 1] for Ni/MoS_2_ and Ni/WS_2_ composites, which further caused the surface texture to change [9], [14] and the surface energy to be minimised.

The full-width half maximum (FWHM) of all moulds was used to calculate the crystallite sizes based on Scherrer’s Equation:

(1) $$D=\frac{K\lambda }{FWHM\;cos\;\theta }$$

where D is the average crystallite size, K and λ are the Scherrer constant (0.89) and the X-ray wavelength (0.154056 nm), respectively. FWHM and θ are the full-width half maxima (in radians) and the corresponding peak position (in radians), respectively. The broadening of FWHM is responsible for the refinement of the average crystallite size. As shown in Fig. 11(b), the FWHM of Ni/MoS_2_ and Ni/WS_2_ composites were broadened compared with that of the pure nickel mould, resulting in the composite moulds having a more refined crystallite size. The FWHM of Ni/WS_2_ composites was the most broadened, suggesting that WS_2_ nanosheets play the most significant role in refining the crystallite size of the composite mould. The refined crystallite size of the composite moulds was due to the following reasons: (i) dispersion effect resulting from the uniformly distributed MoS_2_/WS_2_ nanosheets on the composite mould [9], [14], as proved by the surface morphology described in Section 3.3.1; this dispersion effect is due to the synergistic effect of surfactants CTAB and SDS, and regular ultrasonic treatment; (ii) as MoS_2_/WS_2_ nanosheets were homogeneously dispersed in the solution and then embedded to the nickel deposits, they offered more nucleation sites for nickel atoms by increasing the cathode surface area, thus reducing the grain size of the nickel deposits [14], [53]; (iii) MoS_2_/WS_2_ nanosheets had a “blocking effect” to hinder the growth of nickel atoms, refining the crystallite size [9], [14], [24]. The detailed analysis and discussion of the crystallite size of composite moulds is described below in Section 3.3.3.

#### 3.3.3 Crystallite size and microhardness analysis

**Fig. 12:**
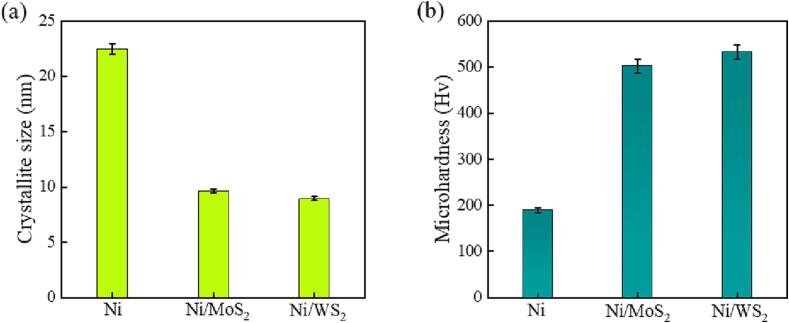
Average crystallite size (a) and microhardness (b) of pure nickel mould, Ni/MoS_2_ and Ni/WS_2_ nanocomposite moulds.

The pure nickel mould had an average crystallite size of 22.45 nm and were relatively soft, with a microhardness of 190 Hv (Fig. 12). With the incorporation of MoS_2_/WS_2_ nanosheets, the crystallite sizes of both composite moulds were significantly reduced, with a decrease of 57% to 9.63 nm for Ni/MoS_2_ and 60% to 8.96 nm for the Ni/WS_2_ composite mould, respectively (Fig. 12(a)). Correspondingly, the microhardness of the composite moulds has been greatly improved due to the incorporated nanosheets. The maximum microhardness of 532 Hv was achieved in the Ni/WS_2_ nanocomposite mould (2.8 times improvement), and the Ni/MoS_2_ composite mould had an enhanced microhardness of 502 Hv (2.6 times increase). The microhardness enhancement of the composite mould occurred for the following reasons: the dispersion effect of the homogenously embedded nanosheets [14]; the strengthening effect of these inert nanosheets [54]; the refined crystallite size [14], [24]; and the hard nature of WS_2_ nanosheets [14], [24]. Here, the peak intensity of [2 0 0] of pure nickel was the highest (Fig. 12 (b and c)), followed by the Ni/MoS_2_ and Ni/WS_2_ composite moulds. However, the high peak intensity of [2 0 0] did not lead to higher microhardness, since the hard nature of WS_2_ nanosheets and refined crystallite size dominated in the hardness enhancement, as also reported in other research [10]. The highest microhardness was associated with the Ni/WS_2_ nanocomposite mould.

#### 3.3.4 Surface roughness and wettability analysis

During the demoulding of micro injection moulding or micro hot embossing, the friction and adhesion forces are the main causes of demoulding defects [6]. The mould should have a low surface roughness (Sa) to reduce mechanical interlocking between the polymer chip and the mould, as a high Sa results in high friction forces during the relative movement of the chip and mould [12]. Meanwhile, a hydrophobic mould surface with low surface energy is favourable for reducing adhesion during demoulding [6], [12]. Therefore, a mould should have low surface energy and low Sa to ensure ease of demoulding.

**Fig. 13:**
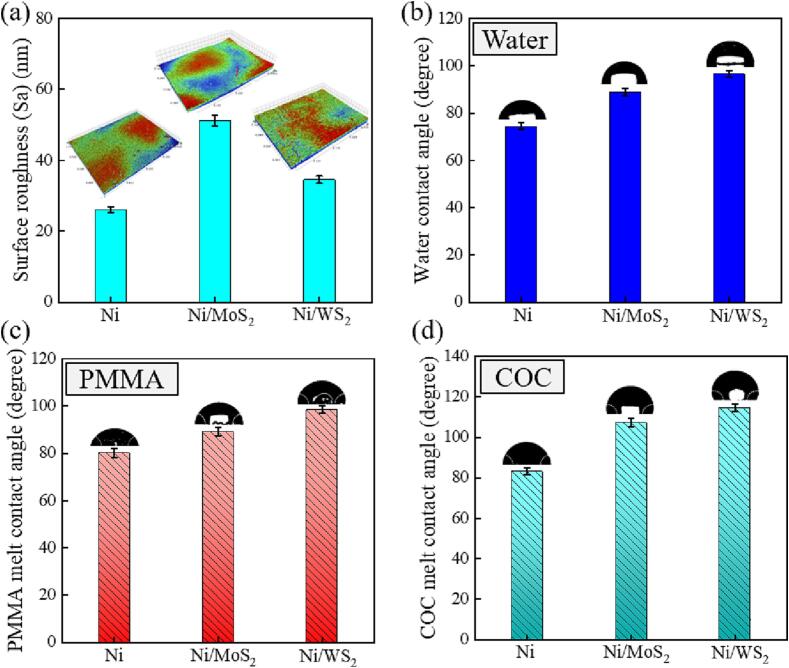
Surface roughness (a), water contact angle (b), PMMA melt contact angle (c) and COC 8007 contact angle (d) of pure nickel mould, Ni/MoS_2_ and Ni/WS_2_ nanocomposite moulds.

Fig. 13(a) shows the Sa of the pure nickel mould and the Ni-MoS_2_/WS_2_ nanocomposite moulds. All moulds had a Sa lower than 60 nm, indicating mirror-like surfaces. The pure nickel mould had a high-gloss surface with a Sa of 26.12 nm. With the incorporation of MoS_2_/WS_2_ nanosheets, the mould surface textures changed due to the shifted preferable orientation from peak [2 0 0] to peak [1 1 1], resulting in higher surface roughness [10], [14]. The Ni/MoS_2_ composite mould had a Sa of 51.25 nm, higher than that of Ni/WS_2_ (34.60 nm), indicating that the Ni/WS_2_ composite mould had a surface with a higher gloss. This could be explained by the better dispersion effect of WS_2_ nanosheets caused by more frequent ultrasonic treatment. The WS_2_ nanosheets were more uniformly embedded in the composite mould surface, as proved by the mould surface morphology discussed in Section 3.3.1.

The mould surface wettability is related to its surface topography and surface chemistry [10]. The measured WCA can be significantly affected by the surface topography. According to Wenzel mode, the wettability of a solid is affected by the Sa [12], [55]. Generally speaking, an increase of Sa will result in higher WCA of a hydrophobic surface, suggesting higher hydrophobicity; while an increase of Sa will lead to a lower WCA for a hydrophilic surface, indicating higher hydrophilicity. As shown in Fig. 13(b), the pure nickel mould had a hydrophilic surface with a WCA of 74.46°. This hydrophilic mould surface has been modified via the incorporation of hydrophobic MoS_2_/WS_2_ nanosheets. The Ni/WS_2_ composite mould had a higher WCA of 96.63° (22.17° increase) than the Ni/MoS_2_ composite mould (88.83°, 14.37° increase), and it also had a lower Sa, which did not follow the Wenzel mode. This is because the increase of hydrophobicity is affected by two factors: (i) the altered surface texture by the incorporated nanosheets, represented as the preferred orientation shifting from [2 0 0] to [1 1 1], which indicates lower surface energy; (ii) the hydrophobicity of MoS_2_/WS_2_ nanosheets. Here, compared to MoS_2_, the uniformly embedded WS_2_ nanosheets played a more important role in increasing the hydrophobicity of the composite mould, resulting in higher WCA.

To further simulate the real micro replication process for polymer materials, the wettability of polymer melt on the mould surfaces was characterized using PMMA and COC 8007 materials, which are commonly used for the fabrication of microfluidic chips, micro-optical elements and photonic devices. Of all the moulds, it was the Ni/WS_2_ composite mould that had the highest hydrophobicity with the polymer melt, having a CA of 98.36° with PMMA melt and a CA of 114.53° with COC melt, potentially reducing the adhesion force at the polymer-mould interface (Fig. 13(c) and (d)). In contrast, the pure nickel mould had a CA of 80.21° with PMMA and 83.19° with COC, indicating the higher surface energy with polymer melts, and the likelihood of higher adhesion forces between the polymer and mould during demoulding and, consequently, more adhesion-induced defects. The Ni/MoS_2_ composite mould had a CA of 89.03° with PMMA and 107.22° with COC, suggesting a lower surface energy with the polymer melt, and consequently less adhesion during demoulding. It should be noted that the COC melt CA was higher than the PMMA melt CA on all nickel mould surfaces, indicating that the adhesion force would be higher at the PMMA-mould interface than at the COC-mould interface. In conclusion, the Ni/WS_2_ nanocomposite mould had a low Sa and high CA with the polymer melt due to its hydrophobic surface, which would serve to reduce demoulding defects, including both friction-induced defects (less mechanical interlocking due to low Sa) and adhesion-induced defects (low surface energy).

#### 3.3.5 Tribological properties

##### 3.3.5.1 Friction tests

**Fig. 14:**
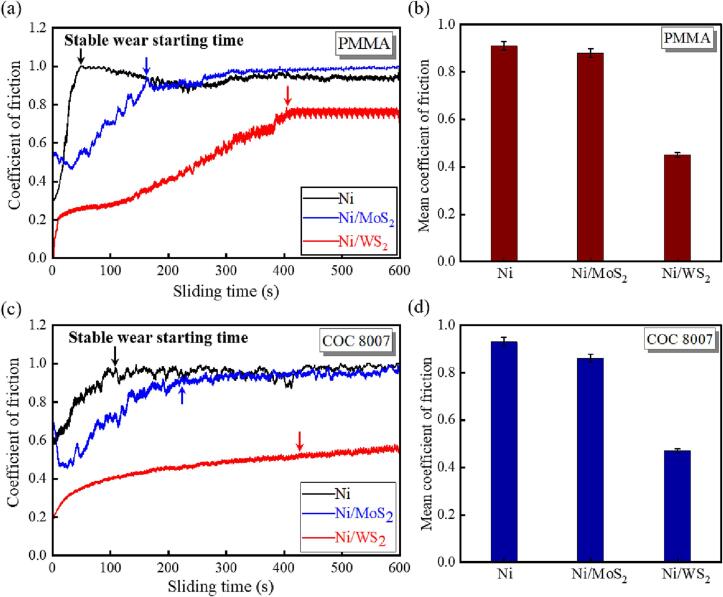
COF vs. sliding time of pure nickel mould, Ni/MoS_2_ and Ni/WS_2_ nanocomposite moulds against the PMMA pin (a) and the mean COF in 600 s (b); the COC 8007 pin (c) and the mean COF in 600 s (d).

Friction-induced surface damage and adhesion-induced distortion are the main causes of demoulding defects, which result in poor surface quality and structural integrity of the polymer chips [6]. Meanwhile, due to the accumulation of adhering polymer and the presence of micro-cuts on the nickel mould surface during demoulding, the tool lifetime of such a mould would be limited by its low wear resistance to polymer material [10]. Therefore, to evaluate the friction and adhesion between polymer and a nickel mould, pins made of PMMA and COC 8007 were used in the pin-on-disc test to simulate the demoulding of polymer from a nickel mould. Fig. 14 shows the relationship between the coefficient of friction (COF) and sliding time, as well as the mean COF for the entire sliding process. Three main important parameters can be obtained for the evaluation of moulds’ lubricating properties:

(i) the initial COF value, which is dependent on the mould surface chemistry and Sa [56]. High Sa results in high mechanical interlocking, and thus a high friction force. An inert and hydrophobic surface indicates low surface energy, leading to low initial COF. The pure nickel mould had an initial COF of 0.31 with PMMA and 0.58 with COC (Fig. 14(a) and (c)). This high initial COF was mainly due to the high surface energy of the pure nickel mould with the polymer material. The Ni/MoS_2_ composite mould had an initial COF of 0.56 with PMMA and 0.65 with COC. This high initial COF was due to the high Sa of the Ni/MoS_2_ composite moulds, leading to more mechanical interlocking and thus higher friction at the beginning of the sliding. The initial COF of the Ni/WS_2_ composite mould was 0.07 for PMMA (0.24 decrease) and 0.21 for COC (0.37 decrease). This major reduction of the initial COF of the Ni/WS_2_ composite mould was mainly due to its low surface energy and low Sa compared with that of the pure nickel mould, leading to both low adhesion and low friction at the beginning of the sliding.

(ii) the stable wear starting time. Two stages existed in the entire sliding process: the initial stage and the steady stage. The stable wear starting time was the setpoint of the steady stage. During the initial stage of PMMA and COC pin-on-disc tests, the COF of pure nickel mould increased rapidly, while the increase rate was much lower for Ni/MoS_2_ and Ni/WS_2_ composite moulds (Fig. 14(a) and (c)), indicating the improved lubricating properties of the composite moulds. After the initial stage, the COF fluctuated and remained stable around a value until sliding ended. The mould surface experienced adhesive wear due to high adhesive forces between the polymer and mould, which was characterized by the polymer material transfer to the mould surface, after which the mould underwent a stationary stage with a stabilized COF and limited wear [57]. Therefore, entering the steady stage suggests the mould surface had already experienced significant wear, which was also indicated by our previous study [10].

In this present study, the duration before stable wear starts is critical in order to estimate the mould tool lifespan [10]. The stable wear starting time (duration) of each mould is indicated by arrows in Fig. 14(a) and (c). The pure nickel mould had a short duration of 51.2 s against PMMA, suggesting it underwent rapid wear during sliding. A decline of COF against PMMA was observed after the stable wear started, which was due to the formation of the protective transfer layer on the pure nickel mould surface, thus decreasing the COF. The Ni/MoS_2_ composite mould had a longer duration of 164.6 s (3.2 times increase), suggesting it would have an extended tool lifetime. A slight decay was observed on the COF curve, indicating the formation of the transfer layer. The Ni/WS_2_ composite mould had the longest tool lifetime with the duration increasing to 408.6 s (8 times increase). There was no decline of its COF curve, indicating that the PMMA protective transfer layer did not develop on its surface.

As for the test with the COC pin, the pure nickel mould had a duration of 108.8 s with a slight decline of COF at the stable wear starting time, indicating the formation of a COC transfer layer. Ni-MoS_2_ had an extended tool lifetime with an increased duration of 227.2 s (2.1 times increase). No significant decline was observed on the COF curve of the Ni/MoS_2_ composite mould, suggesting that no continuous COC transfer layer was formed on its surface. The Ni/WS_2_ composite mould had the longest duration of 449.6 s (4.2 times increase). A smooth COF curve without any peak was observed for the Ni/WS_2_ composite mould, suggesting the absence of a COC protective transfer layer. In conclusion, the incorporation of MoS_2_/WS_2_ nanosheets was effective in extending the lifetime of the composite mould. WS_2_ nanosheets played the most critical role in extending mould lifetime by reducing the mould surface energy and increasing its hardness: this was evidenced by the longest duration before the mould experienced stable wear using both the PMMA and COC pins.

(iii) the mean COF, indicates the overall effective lubricating properties [10]. As shown in Fig. 13(b) and (d), the incorporation of MoS_2_/WS_2_ nanosheets was effective in reducing the average COF of the composite moulds. The mean COF with PMMA reduced from 0.91 for pure nickel to 0.88 for Ni-MoS_2_ and further to 0.45 for Ni/WS_2_ (51% reduction). Similarly, the mean COF with COC also decreased from 0.93 for pure nickel to 0.86 for Ni/MoS_2_ and to 0.47 for Ni/WS_2_ (49% reduction). The Ni-MoS_2_ composite mould had a relatively high mean COF, mainly due to its high Sa, causing high friction. The significant decrease of the mean COF for the Ni/WS_2_ composite mould was mainly due to three reasons: high microhardness, indicating less real contact area [16], [58]; low surface energy, indicating less adhesion [14]; low surface roughness, suggesting less mechanical interlocking [6]. In conclusion, the Ni/WS_2_ composite mould had the lowest COF and longest tool lifetime of all moulds, which indicated reduced friction- and adhesion-induced defects would occur during demoulding.

##### 3.3.5.2 Wear morphology analysis

**Fig. 15:**
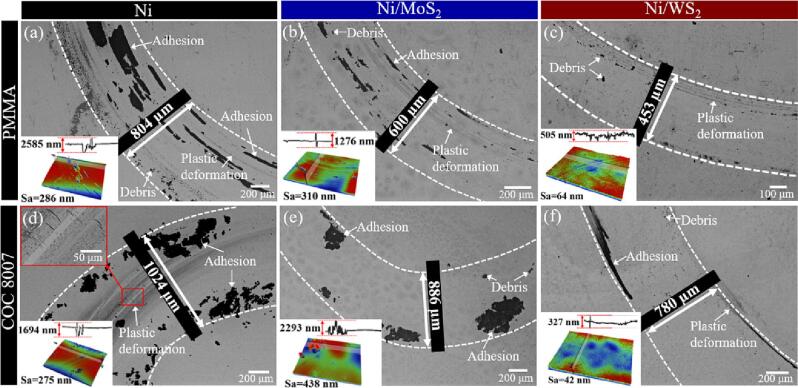
SEM images and surface profile of wear morphology on the moulds against the PMMA pin (a ∼ c) and the COC 8007 pin (d ∼ f): pure nickel mould (a and d), Ni/MoS_2_ mould (b and e) and Ni/WS_2_ mould (c and f) after the pin-on-disc test.

The surface morphology of the wear track is critical to determine the wear resistance of a mould against the polymer material [14]. The wear morphology and topography are shown in Fig. 15. When tested with a PMMA pin, adhesion-dominated wear and plastic deformation-induced wear were found on the pure nickel mould surface with a wear track of 804 µm (Fig. 15(a)). Continuous PMMA transfer film developed on the mould surface. The Sa increased from 26.12 nm to 286 nm after wear, mainly caused by adhesive wear, as also proven by the wear topography. As for the test with the COC pin, severe plastic deformation-induced wear with micro-cracks was detected on the wear track, and adhesion-dominated wear was found on the pure nickel mould surface (Fig. 15(d)). The wear track width was 1024 µm, with the Sa increasing to 275 nm after the friction test. The development of the PMMA transfer film and COC clumps was mainly due to the high surface energy of the pure nickel mould, causing severe adhesive wear during the pin-on-disc tests. The microhardness of a metal can be used to describe its relative ability to resist scratching or abrasion [59]. Therefore, the low microhardness of the pure nickel mould caused it to suffer severe plastic deformation-induced wear caused by micro-cutting or micro-scratching from the polymer pin. The high surface energy and low microhardness of the pure nickel mould would accelerate the frequency of replacing such a mould insert because of micro-cuttings from polymer chips, and the accumulation of polymer adhesion occurring on the mould surface, both of which would deteriorate the mould surface during repeated moulding cycles.

Adhesion-dominated wear caused by the PMMA and COC pins has been largely decreased with the Ni/MoS_2_ composite mould, as evidenced by the reduced wear track width of 600 µm and 886 µm against the PMMA and COC pin, respectively (Fig. 15(b) and (e)). However, the Sa of the Ni/MoS_2_ composite mould after wear increased significantly from 51.25 nm to 310 nm against the PMMA pin and to 438 nm against the COC pin. This was because the Sa of the Ni/MoS_2_ composite mould was high, causing more mechanical interlocking during the polymer pin’s sliding. During sliding, a small quantity of debris was first cut from the polymer pin and transferred to the mould surface. This debris then aggregated and accumulated on the mould surface and developed into clumps and further into transfer layers, depending on the mould surface energy and Sa. Higher Sa levels caused more debris to be removed from the polymer pin, while higher surface energies allowed polymer clumps or even polymer transfer layer to form on the mould surface. Ni-MoS_2_ had a WCA of ∼88.83°, which was more hydrophobic than the pure nickel mould (WCA of 74.46°). However, this surface energy was not low enough to prevent the formation of polymer clumps, and the accumulation of polymer clumps resulted in high Sa after wear, as confirmed by the wear topography (Fig. 15(e)).

Among these moulds, it was the Ni-WS_2_ composite mould that had the highest wear resistance against both the PMMA and COC pins, represented as the very mild plastic deformation-induced wear and very small debris and significantly reduced adhesive wear on the mould surface (Fig. 15(c) and (f)). The wear track on the Ni-WS_2_ composite mould was reduced to 453 µm and 780 µm against the PMMA and COC, respectively. Most importantly, the Sa was well maintained after wear, with a slight increase to 64 nm and 42 nm against PMMA and COC, respectively. The height of the microgroove on the wear track was reduced by 80.46% from 2585 nm for the pure nickel mould against PMMA to 505 nm for the Ni/WS_2_ composite mould and was reduced by 80.69% from 1694 nm for pure nickel to 327 nm for Ni/WS_2_ against COC.

The significantly enhanced wear resistance of the Ni/WS_2_ composite mould against the polymer pin was mainly due to three reasons: (i) low surface energy, indicating less adhesion at the polymer-mould interface during sliding [10], [14]; (ii) high microhardness, representing a higher ability to resist micro-scratching or micro-cutting from the polymer pin [10], [14], [48], [50], [59]; (iii) low original Sa, causing less mechanical interlocking during sliding [6], [14]. The hydrophobicity of the Ni/WS_2_ composite mould was the most influential factor in reducing the adhesive wear, as the composite mould with the lowest surface energy inhibited the formation of a robust and continuous polymer transfer layer, which was consistent with the findings of our previous study [14]. Consequently, it can be concluded that a hydrophobic mould surface will ease the process of mould release by minimising adhesion to a polymer workpiece.

### 3.4 Characterization of hot-embossed polymer chips

**Fig. 16:**
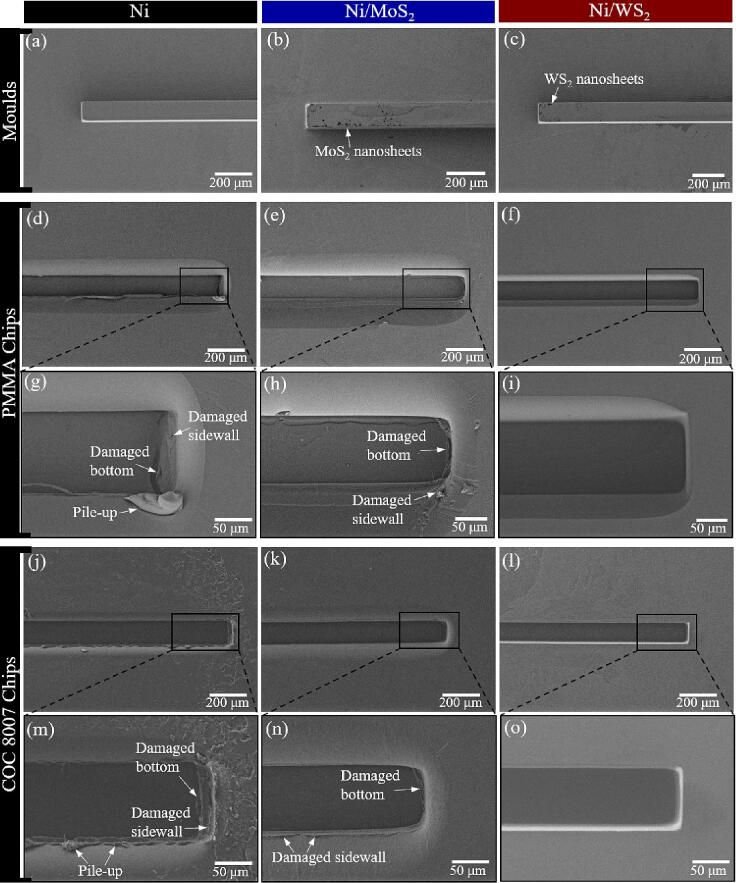
The surface morphology of ridges in the moulds with a width of 100 µm and a height of 100 µm (a ∼ c); the surface morphology of the channels in the hot-embossed PMMA chips at lower magnification (d ∼ f) and higher magnification (g ∼ i); the surface morphology of the channels in the hot-embossed COC 8007 chips at lower magnification (j ∼ l) and higher magnification (m ∼ o).

Fig. 16(a ∼ c) shows the micro features on the mould surfaces. All electroformed moulds had good surface quality and structural integrity, with MoS_2_ and WS_2_ nanosheets being detected on the composite mould surfaces. The micro ridges had a width of 100 µm and a height of 100 µm. After micro hot embossing, the replicated PMMA and COC 8007 chips were also characterized to compare any demoulding defects.

The defects found on the PMMA chips that were embossed from the pure nickel mould included damage to the sidewall and bottom and a significant quantity of piled-up polymer. These defects were caused by high friction forces and high adhesion forces, respectively (Fig. 16(d) and (g)). The COC chip embossed from the pure nickel mould also had similar demoulding defects that were caused by high friction and high adhesion at the COC-Ni mould interface (Fig. 16(j) and (m)). These demoulding defects were mainly due to the high COF and high surface energy of the pure nickel mould.

In comparison, the pile-up defects on the chips that were embossed from the Ni/MoS_2_ composite mould have been largely reduced in size, due to the lower adhesion at the polymer-mould interface. However, due to the high original Sa and high COF of the Ni/MoS_2_ composite mould, broken edges and some damage to the bottom and sidewall regions still occurred on the embossed PMMA and COC chips (Fig. 16(e), (h), (k) and (n)). The reduced adhesion was due to the reduced surface energy that resulted from the incorporated MoS_2_ nanosheets. Clearly, a mould should also have low COF and Sa in order to avoid high friction forces during demoulding.

The final set of results show that both of the PMMA and COC chips that were embossed using the Ni/WS_2_ nanocomposite mould presented the best demoulding results, namely, a neat surface with good structural integrity. Channels on these chips had sharp edges without any pile-up defects, as a consequence of the significantly reduced adhesion at the polymer-mould interface. Meanwhile, no surface damage was detected on the chip surfaces, suggesting low friction as well. In conclusion, the superior ability of the Ni/WS_2_ composite mould in easing mould release can be attributed to its low COF (low friction force) and high surface energy (low adhesion force), which was due to the uniformly incorporated WS_2_ nanosheets in the nickel. Meanwhile, the high microhardness of the Ni/WS_2_ composite mould prevented it from severe wear caused by abrasion from the polymer workpieces, extending the mould lifetime.

A brief cost assessment for producing Ni-MoS_2_/WS_2_ nanocomposite moulds can be achieved by calculating the consumption of materials including surfactants and nanomaterial dispersions that were used to fabricate each of the two composite moulds. Fabricating one 4-inch pure nickel mould cost ∼4000 Euro (manufacturing setup and material costs including patterned silicon wafer fabrication, wafer surface metallization and electroforming). MoS_2_/WS_2_ nanosheet dispersions cost ∼500 Euro/500 mL; each nanocomposite mould consumed ∼100 mL dispersions. Thus, to fabricate one composite mould, the cost of nanomaterials was ∼100 Euro. Additionally, the cost of surfactants CTAB and SDS was ∼60 Euro/100 g. For each nanocomposite mould, less than 2 g of surfactants were used, corresponding to a cost of ∼1.2 Euro. In summary, the fabrication of one nickel-MoS_2_/WS_2_ nanocomposite mould cost 4101.2 Euro, which corresponded to a 2.53% increased cost of the pure nickel mould (i.e., an extra 101.2 Euro). This up-front cost does not reflect the operational costs associated with the three moulds. Compared with the pure nickel mould, the tool lifetime of the Ni-MoS_2_/WS_2_ nanocomposite moulds were extended significantly, by factors of 3.2 times and 8 times, respectively. Additionally, the composite moulds have the inherent advantage of causing fewer or zero demoulding defects, avoiding the cost of applying lubricating coatings to a mould insert. Such coatings are expensive both initially and repeatedly as they delaminate during micro-injection moulding/hot embossing. Consequently, in terms of the total tool life costs associated with producing a large quantity of moulded or embossed polymer components, the use of nickel-MoS_2_/WS_2_ nanocomposite moulds is competitive and cost-effective. Therefore, we believe that the fabrication process for nickel-based nanocomposite moulds can be easily scaled in a way that is compatible with existing industrial low-cost production processes. This offers significant potential to produce large quantities of polymeric micro/nano devices that are both cost-effective and defect-free.

## 4 Conclusion

In this work, we studied the synthesis of 2D material MoS_2_/WS_2_ nanosheets with surfactants under ultrasonication. The effect of ultrasonic power, processing time, surfactant types and concentrations on the properties of nanosheets was studied to elaborate their dispersion mechanism and control their size and surface charge in divalent nickel electrolytes. The formulation of 2D MoS_2_/WS_2_ nanosheets was then optimized for effective electrodeposition of nickel nanocomposite with enhanced hardness without overwhelming electrophoresis deposition and adsorption. A novel strategy of intermittent ultrasonication in the dual bath was then developed to overcome the overheating of electrolytes from continuous ultrasonication for long-term electroforming of nickel nanocomposite mould. 4-inch wafer-scale Ni-MoS_2_/WS_2_ nanocomposite moulds were thus fabricated to validate this strategy. The results demonstrated that the surface properties and tribological performance were significantly improved due to the MoS_2_/WS_2_ nanosheets being uniformly dispersed. The main conclusions of this study can be summarised as follows:

(1) The mixture of surfactants CTAB and SDS had a synergistic effect and effectively reduced the size of the MoS_2_ and WS_2_ nanosheets to ∼100 nm and produced a narrow particle size distribution with a PDI of ∼0.2 in the divalent nickel sulphamate solution. Catatonic-rich surfactants modified the nanosheets to have a controlled net positive charge, facilitating their electrophoresis to the nickel deposit without aggressive adsorption.

(2) The use of medium ultrasonic power (60%) and treatment time (20 min) proved effective in dispersion of these nanosheets in the nickel sulphamate solution, and ensured the smallest nanosheet size (104 nm for MoS_2_ and 106 nm for WS_2_) and PDI (∼0.2), which indicated uniform particle distribution of these nanosheets in the electrolyte. The settling time was detected as being 25 min and 15 min, respectively, for the MoS_2_ and WS_2_ nanosheets before starting a second ultrasonic treatment to prevent the reaggregation of these nanosheets during electroforming.

(3) The electroformed Ni-WS_2_ composite mould using optimized formulation and ultrasonication process showed the best lubrication properties and significantly reduced COF (from 0.91 to 0.45 and from 0.93 to 0.47 against PMMA pin and COC pin, respectively). Compared with a pure nickel mould, the incorporation of MoS_2_ and WS_2_ extended the mould tool lifetime by 3.2 times and 8 times, respectively, with enhanced wear resistance against a polymer pin. The microhardness has been improved by 2.6 and 2.8 times for Ni-MoS_2_ and Ni-WS_2_ composite moulds, respectively. The mould surface has been tuned from the hydrophilicity of pure nickel to being hydrophobic with the WCA increasing from 74.46° for pure nickel to 88.83° for Ni-MoS_2_ and 96.63° for Ni-WS_2_.

(4) The PMMA and COC 8007 chips were embossed using the Ni-MoS_2_/WS_2_ composite moulds, which had significantly fewer surface and structural defects.

## CRediT authorship contribution statement

**Tianyu Guan:** Conceptualization, Methodology, Software, Data curation, Writing – original draft. **Yuanzhi Lu:** Methodology. **Xinhui Wang:** Software. **Michael D. Gilchrist:** Writing – review & editing. **Fengzhou Fang:** Writing – review & editing. **Nan Zhang:** Conceptualization, Resources, Supervision, Writing – review & editing.

## Declaration of Competing Interest

The authors declare that they have no known competing financial interests or personal relationships that could have appeared to influence the work reported in this paper.

## Data Availability

Data will be made available on request.
